# Regulatory, ethical, and clinical barriers to exosome use in interventional pain medicine

**DOI:** 10.1016/j.inpm.2026.100746

**Published:** 2026-03-03

**Authors:** Alexandre J. Bourcier, Zbigniew M. Kirkor

**Affiliations:** a Department of Medicine, University of Pittsburgh Medical Center, Pittsburgh, PA, USA; b Algocells Clinic, London, UK; c Specialist Hospital Matopat, Torun, Poland

## Abstract

Exosome-based therapies have drawn growing attention in interventional pain medicine as clinicians look for regenerative options beyond corticosteroids, radiofrequency ablation, and cell-based procedures. The early preclinical work suggests anti-inflammatory and neuromodulatory effects relevant to muskuloskeletal and spine pain. However, this promise is currently outpaced by clinical marketing. No exosome product is United States Food and Drug Administration (FDA) approved for musculoskeletal or pain indications, and all therapeutic use in the United States requires an Investigational New Drug application, Institutional Review Board oversight, and Good Manufacturing Practice (GMP) grade manufacturing. Despite this, a parallel cash-pay market has expanded, offering “361-compliant” or “minimally manipulated” exosomes for intradiscal, epidural, or joint injection, in direct conflict with FDA guidelines.

This review outlines the regulatory landscape governing exosome products in the United States, Europe, and Asia; describes key manufacturing and characterization challenges that undermine consistency and potency; and examines the ethical and medico-legal issues created when unapproved biologics are marketed directly to patients. Scientific barriers remain substantial, including heterogeneous isolation techniques, unstable product profiles, unresolved dosing metrics, and limited human data. These gaps, combined with high economic barriers to GMP production, prevent exosomes from being responsibly incorporated into interventional pain practice.

## 1 Introduction

Interest in regenerative biologics has rapidly expanded across interventional pain medicine. This shift is driven in part by frustration with the limited durability of steroid injections, the risks and diminishing returns of repeated ablative procedures, and a desire for definitive treatment of the root cause of pain rather than muting its symptoms as recently mentioned by Robinson and colleagues [[1], [2], [3]]. Platelet-rich plasma (PRP), mesenchymal stem cell (MSC) preparations, and amniotic products have all entered the musculoskeletal and spine space with varying levels of evidence and regulatory clarity over the last few years [[1], [2], [3], [4], [5], [6]]. Exosomes and other extracellular vesicles have now been added to that list.

Exosomes are small extracellular vesicles that carry proteins, lipids, cytokines, and ribonucleic acid (RNA) cargo released by parent cells into the extracellular space. They can regulate inflammation, influence immune cell behavior, and support tissue repair in preclinical models [[7], [8], [9], [10]]. Exosome preparations derived from MSCs and other cell types have shown encouraging effects in models of disc degeneration, facet joint inflammation, tendon injury, and neuropathic pain [[7], [8], [9], [10]]. A recent review summarized early exosome work in low back pain and concluded that the field is promising but preliminary, with almost all evidence coming from animal studies and a handful of small human reports [8]. Despite those findings, exosome products are already being promoted in clinical practice.

Clinics across the United States advertise “exosome injections” for back pain, arthritis, neuropathy, and systemic conditions. Marketing language frequently suggests that these products are “FDA registered,” “361 compliant,” or “minimally manipulated,” implying a level of regulatory acceptance that does not exist [[11], [12], [13], [14]]. The FDA has issued a public safety notification on exosome products, a consumer alert on regenerative medicine therapies that include exosomes, and warning letters to specific companies for marketing unapproved exosome products associated with adverse events [[11], [12], [13], [14]]. This gap between research progress and real-world use raises predictable concerns. Enthusiasm for a mechanistically attractive biologic is running ahead of regulatory pathways, manufacturing standards, and controlled clinical trials. For interventional pain physicians, this creates a tension as exosomes may eventually become part of a regenerative toolkit, but current use typically does not follow proper regulation.

The purpose of this review is to describe the regulatory framework that governs exosome products, review the scientific and clinical barriers that limit their current use, and highlight ethical, marketing, and economic issues that are particularly relevant to interventional pain practice. The intent is to help clinicians stay grounded in what is known, what is not, and what is required before exosomes can be responsibly integrated into musculoskeletal and spine care.

## 2 Regulatory framework

### 2.1 U.S. FDA pathway

In the United States (US), exosome products fall under the FDA's framework for Human Cells, Tissues, and Cellular and Tissue-Based Products (HCT/Ps). These products are regulated either under Section 361 of the Public Health Service Act or as biologic drugs under Section 351. To qualify as a 361 HCT/P, a product must meet several criteria, including minimal manipulation, homologous use, and lack of systemic effect or dependence on the metabolic activity of living cells [4]. By fulfilling the above criteria, 361 products do not require FDA premarket approval, though they must still follow good manufacturing and infection control practices.

Exosome preparations do not fit within this 361 category. They are not a tissue in the traditional sense, they are isolated and processed using laboratory methods, and their intended effect is to modulate inflammation, promote regeneration, or alter neural signaling in a way that is not homologous to the original source tissue [[7], [8], [9]]. For these reasons, exosome products are classified as Section 351 biologic drugs that require an Investigational New Drug (IND) application for clinical trials and a Biologics License Application (BLA) for marketing approval [4].

In 2019, the FDA issued a Public Safety Notification on exosome products after serious adverse events were reported in patients who received unapproved preparations marketed as exosomes [11]. In 2020, a Consumer Alert on regenerative medicine products, including stem cells and exosomes, reminded clinicians and patients that these products are regulated as drugs, biologics, or medical devices and generally require FDA approval before marketing [12]. More recently, warning letters have been sent to companies and clinics selling “umbilical cord–derived exosomes” and similar products without an IND or BLA, citing them as unapproved new drugs and unlicensed biological products [13,14]. Concerns raised in those letters include manufacturing outside of Good Manufacturing Practice (GMP) standards, lack of validated sterility and potency testing, and promotional claims that a product is safe and effective for conditions such as muskuloskeletal pain, spine disorders, or systemic inflammation without adequate evidence [13,14].

### 2.2 International regulatory landscape

Outside the US, regulatory authorities adopt an equally conservative stance toward exosome-based therapies. In the European Union (EU), exosome products intended to exert pharmacologic, immunologic, or metabolic effects are generally regulated as Advanced Therapy Medicinal Products (ATMPs) under Regulation (EC) No 1394/2007, requiring centralized European Medicines Agency (EMA) authorization, full Chemistry, Manufacturing, and Controls (CMC) documentation, GMP-compliant manufacturing, as well as evidence from controlled clinical trials [15]. Japan typically classifies extracellular vesicle (EV) products without viable cells as drugs rather than regenerative medical products, subject to conventional Pharmaceuticals and Medical Devices Agency (PMDA) review with the possibility of conditional, time-limited approval based on early efficacy and safety data [15]. South Korea's Ministry of Food and Drug Safety (MFDS) has issued EV-specific guidelines that similarly mandate defined source materials, validated isolation and characterization methods, nonclinical pharmacology and toxicology, and formal clinical studies within a GMP framework [15].

For interventional pain practice, the implications are direct. In the US, any clinically legitimate use of an exosome product must occur under a bona fide FDA IND application, tied to a specific manufacturing process and indication, overseen by an IRB, and supported by GMP manufacturing with predefined release specifications and batch-level sterility and quality testing. In the absence of these features, the material in use is an unapproved biological drug, irrespective of marketing claims. Notably, the concept of “361-compliant” exosomes used for epidural, intradiscal, or intra-articular injection is not supported by current FDA policy and exposes clinicians to substantial regulatory and medico-legal risk.

## 3 Scientific and clinical barriers

Even if the regulatory pathway were clear and uniformly followed, several scientific and clinical obstacles still limit the readiness of exosomes for routine pain practice.

### 3.1 Manufacturing variability and potency

Manufacturing exosome products that are consistent from batch to batch is not trivial. Parent cell type, donor characteristics, culture media, oxygen tension, and preconditioning all influence vesicle content and function [7,9]. Isolation methods vary widely. Differential ultracentrifugation, size-exclusion chromatography, tangential flow filtration, and immuno-affinity capture all generate products with different purity, yield, and co-isolated contaminants [7,9].

The International Society for Extracellular Vesicles (ISEV) has published MISEV2023, an updated set of “Minimal Information for Studies of Extracellular Vesicles” that outlines expectations for vesicle characterization, including particle sizing, marker profiling, and functional assays [16]. Despite this, many studies of exosomes in musculoskeletal or spine disease still report limited characterization, making it difficult to compare products across studies or translate them to clinical-grade manufacturing [17,18].

More importantly for clinicians, there is no agreed-upon potency assay for exosome products in pain applications. Dose is often expressed as particle count or total protein, but these measures may not correlate with biologic activity. Functional assays, such as suppression of inflammatory cytokine production or support of chondrocyte or neuronal survival in vitro, are a step in the right direction but remain heterogeneous and not yet validated as surrogates for clinical outcomes.

### 3.2 Dose definition and delivery

On the clinical side, basic questions about dose and delivery remain unanswered. In preclinical disc and joint models, exosomes have been given at very different particle doses, in varying volumes, with inconsistent timing and repeat schedules [9,10]. Early human studies in low back pain and osteoarthritis use different metrics altogether, which makes it difficult to design rational dose-escalation trials [8,17,18].

Interventional pain procedures add another layer of complexity. Intradiscal injections must contend with a relatively avascular, high-pressure environment where injectate spread and retention are not fully understood. Epidural and perineural injections expose neural tissue directly to exosome products, raising theoretical concerns about aberrant sprouting or unintended neuroimmune effects. Facet and sacroiliac joint injections place exosomes into a synovial environment where rapid clearance is likely. Tendon and ligament injections raise questions about how exosomes behave under mechanical loading. None of these issues are insurmountable, but they highlight how early the field still is.

### 3.3 Clinical evidence gap

From an evidence standpoint, exosome therapies for low back pain and related conditions are at the very beginning of the clinical curve. There are only a few clinical studies most of which are uncontrolled, small, and methodologically heterogeneous [9]. By comparison, PRP and MSC-based approaches, while still debated, now have multiple randomized or at least controlled studies in discogenic and facet-related pain [1,6]. Until exosome products are tested in well-designed, adequately powered trials with clearly defined endpoints and standardized products, any claim of clinical benefit in pain or spine indications should be viewed as hypothesis-generating rather than practice-changing.

## 4 Ethical, marketing, and economic concerns

The ethical issues surrounding exosome therapy in pain medicine overlap with those already seen in stem cell and other regenerative products, but with a few new twists. Many cash-pay clinics advertise “stem cell exosomes” or “exosome regenerative injections” as treatments for back pain, arthritis, neuropathy, or systemic inflammation [[11], [12], [13], [14]]. Online content often blends preclinical data, anecdotal case reports, and generic regenerative medicine language in a way that can be difficult for patients to interpret. Regulatory terms are frequently misused, with claims that a product is “FDA registered” or “361 compliant” presented as evidence of approval or safety.

The FDA and professional societies have repeatedly warned that such marketing can be misleading and potentially dangerous [[11], [12], [13], [14]]. Patients may reasonably assume that therapies advertised by licensed clinics have been vetted to the same standard as approved drugs, when in reality they are receiving products that have not been formally evaluated for safety, dose, or efficacy.

Informed consent in this context is often inadequate. If the investigational nature of the product, the lack of regulatory approval, and the uncertainty around long-term risk are not clearly explained, patient autonomy is compromised. The financial dimension is also important. Exosome injections are typically offered as out-of-pocket services at significant cost, which raises questions about equity and expectation management.

For interventional pain physicians, this landscape mirrors the challenges previously seen with amniotic fluid and other biologic injectables. Clinician interest in novel therapies for refractory conditions is understandable. However, there remains a professional obligation to accurately represent the current state of evidence and to avoid being influenced by commercial claims when robust clinical data are lacking.

True clinical development of exosome therapeutics is expensive. GMP-scale manufacturing requires controlled facilities, validated isolation and purification systems, sterility, and stability studies. Analytical assays for identity, purity, and potency add further cost [16]. Early-phase clinical trials in musculoskeletal and spine indications would need to support imaging, laboratory monitoring, long-term follow-up, and centralized data analysis.

At present, there are no FDA-approved exosome indications for any pain or musculoskeletal condition, and no established reimbursement pathway. As a result, legitimate clinical development depends on research funding and industry investment that may be difficult to secure, especially when unregulated cash-pay clinics can market “exosome injections” without bearing the cost of GMP manufacturing, regulatory submission, or rigorous trials. This disconnect between compliant and non-compliant players slows progress for everyone.

## 5 Practical guide for interventionalists

Clinicians navigating the current exosome landscape are best served by grounding every decision in transparency and regulation. Any preparation offered for musculoskeletal or spine indications should come with clear documentation of its origin, manufacturing conditions, and release testing. If a company cannot specify the parent cell line, donor screening, culture environment, or isolation method, or if it cannot provide Certificates of Analysis (CA) that document sterility and particle characterization, that gap alone is enough to question the product's legitimacy. The same applies to regulatory paperwork. In the United States, lawful clinical use requires an active IND, IRB approval, and an identifiable study sponsor. Without these elements, the product is an unapproved biologic, regardless of how often terms like “minimally manipulated,” “361-compliant,” or “FDA registered” appear in marketing copy.

Because exosome therapy for pain is still firmly in the research phase, clinicians should expect evidence to reflect early-phase trial design rather than established clinical benefit. Any investigational use should be publicly registered with clear endpoints and monitoring plans, and informed consent should make it unambiguous that the therapy is unapproved and of uncertain long-term risk and benefit. Red flags such as claims of FDA approval, absence of sterility data, heavy reliance on testimonials, or expensive cash-only offerings outside a formal trial deserve real scrutiny. When those signals appear, the most responsible move is to counsel patients honestly about where the science stands and to steer them toward regulated trials rather than unmonitored commercial injections.

## 6 Conclusion

Exosomes and other extracellular vesicles represent a promising biologic avenue for refractory musculoskeletal and spine pain, with preclinical data and early human reports supporting further investigation. However, their current clinical use lags far behind commercial messaging. Regulatory bodies classify exosomes as biologic drugs, manufacturing standards and potency assays remain under development, and rigorous clinical trials are only beginning. In this setting, premature clinical adoption introduces avoidable risks for both patients and practitioners. For interventional pain physicians, a measured stance is warranted: exosomes should be regarded as investigational rather than established therapies, and enthusiasm should be tempered by adherence to evidence and regulatory discipline.

## Declaration of generative AI and AI-assisted technologies in the writing process

During the preparation of this work the authors used ChatGPT in order to improve writing style and proofread for any inconsistencies. After using ChatGPT, the authors reviewed and edited the content as needed and take full responsibility for the content of the publication.

## Funding

This research did not receive any specific grant from funding agencies in the public, commercial, or not-for-profit sectors.

## Conflicts of interest

None.
